# Green Communication for Underwater Wireless Sensor Networks: Triangle Metric Based Multi-Layered Routing Protocol

**DOI:** 10.3390/s20247278

**Published:** 2020-12-18

**Authors:** Ahmad M. Khasawneh, Omprakash Kaiwartya, Jaime Lloret, Hayfa Y. Abuaddous, Laith Abualigah, Mohammad Al Shinwan, Mahmoud Ahmad Al-Khasawneh, Marwan Mahmoud, Rupak Kharel

**Affiliations:** 1 Department of Mobile Computing, Amman Arab University, Amman 11953, Jordan; a.khasawneh@aau.edu.jo (A.M.K.); haddose@aau.edu.jo (H.Y.A.); laythdyabat@aau.edu.jo (L.A.); mohmdsh@aau.edu.jo (M.A.S.); 2 Department of Computer Science, Nottingham Trent University, Nottingham NG11 8NS, UK; omprakash.kaiwartya@ntu.ac.uk; 3 Department of Communications, Universitat Politècnica de València, Valencia, 46022, Spain; jlloret@dcom.upv.es; 4 School of Computing and Digital Technologies, Staffordshire University, Stoke ST4 2DE, UK; 5 Faculty of Computer & Information Technology, Al-Madinah International University, Kuala Lumpur 57100, Malaysia; mahmoud@outlook.my; 6 Department of Computer and Information Technology, Faculty of Applied Studies, King Abdulaziz University, Jeddah 21589, Saudi Arabia; mmamahmoud@kau.edu.sa; 7 Department of Computing and Mathematics, Manchester Metropolitan University, Manchester M15 6BH, UK

**Keywords:** link quality, pressure sensors, residual energy, sink node, supernodes, triangle metric, underwater wireless sensor network

## Abstract

In this paper, we propose a non-localization routing protocol for underwater wireless sensor networks (UWSNs), namely, the triangle metric based multi-layered routing protocol (TM2RP). The main idea of the proposed TM2RP is to utilize supernodes along with depth information and residual energy to balance the energy consumption between sensors. Moreover, TM2RP is the first multi-layered and multi-metric pressure routing protocol that considers link quality with residual energy to improve the selection of next forwarding nodes with more reliable and energy-efficient links. The aqua-sim package based on the ns-2 simulator was used to evaluate the performance of the proposed TM2RP. The obtained results were compared to other similar methods such as depth based routing (DBR) and multi-layered routing protocol (MRP). Simulation results showed that the proposed protocol (TM2RP) obtained better outcomes in terms of energy consumption, network lifetime, packet delivery ratio, and end-to-end delay.

## 1. Introduction

Lately, underwater wireless sensor networks (UWSNs) have attracted many researchers due to their various applications in different fields such as environmental, scientific, military, and commercial [1,2,3]. In UWSNs, the radio and optical signals affect the network performance, particularly in radio signals, which require large antennas and tremendous transmission energy as they propagate at low frequencies (30–300) and long-distance [4,5,6]. On the other hand, optical signals need high accuracy in aiming the small laser beams and are influenced by scattering [7,8,9]. Therefore, acoustic signals can be used with UWSNs as a communication medium as acoustic signals can overcome these limitations [10,11,12]. Nevertheless, using these acoustic signals, the UWSNs have several challenges such as high propagation delay (about 1500 m/s), insufficient bandwidth (less than 100 KHz), and high bit error rate as a result of the intense aspects of the underwater channel [13,14,15]. Furthermore, in UWSNs, the sensor nodes have low energy with high battery replacement because of the harsh environment [16,17,18]. Consequently, it is necessary to increase the overall network lifetime in UWSNs [19,20,21].

Regarding the challenges mentioned previously, routing protocols in the terrestrial sensor network are not appropriate for underwater areas; instead, numerous algorithms have been suggested for UWSNs [22,23]. The UWSN routing protocols are categorized into localization-based and non-localization protocols, where the localization-based ones need full positioning information using the Global Positioning System (GPS). However, the harshness of the underwater areas decreases the appropriateness of localization-based algorithms in UWSNs. Accordingly, non-localization routing protocols have attracted researchers to investigate UWSNs [24,25,26]. Furthermore, the non-localization protocols undergo an efficient aggregate of metrics, resulting in unstable links and high energy consumption forwarder nodes. The depth-based routing only uses the depth information, which leads to choosing high power consumer nodes as a next-hop forwarder [27,28,29]. energy efficient depth based routing (EEDBR) uses the depth metric and remaining power without link quality, leading to wastage of power in various paths provoked by unbalanced link choice [30].

In related literature, a vector-based forwarding protocol (VBF) strategy has been suggested requiring position information of all nodes in the network [31,32]. Furthermore, it was improved as Hop-by-Hop (HH) VBF, considering the pipelining of positioning information of the forwarding path in the network [33]. Another improvement, vector based void avoidance (VBVA) utilizes the 3D flooding approach for detecting empty areas in the network [34]. A cross layer focused beam routing (FBR) has been suggested as a beaconing centric improvement relying on request to send and clear to send overhead packets [35,36]. Similarly, location free approaches [37,38,39,40] have utilized expected transmission cost (ETX) as major network monitoring parameters, which are not practically suitable in most underwater networking applications [41].

Therefore, considering green computing concepts in underwater wireless sensor networks, it is necessary to design and develop an efficient algorithm that handles the problems above-mentioned effectively to reduce energy consumption, improve the packet delivery ratio, and further maximize the network lifetime. In this paper, we introduce a non-localization routing protocol for UWSNs, called the triangle metric based multi-layered routing protocol for UWSNs (TM2RP). Moreover, we employed an energy balancing technique to improve the network lifetime. The main aim of the proposed TM2RP is to utilize a set of supernodes as a leading part of the forwarding process. We want to clarify that our depth based underwater networking architecture is computationally novel and capable of saving more energy than the literature architecture presented in MRP [42]. We utilized a supernode to identify a one-layer of nodes among supernodes. The nodes placed in the identified layer are called neighbor of supernode (NSN), which can forward the data packet directly to the supernode, reducing retransmission and overall energy consumptions. Moreover, supernodes are responsible for determining the NSN. The one-layer of nodes also reduces networking overheads compared to the architecture with several layers of node identification. On the other hand, in MRP [42], supernodes have been utilized to identify several layers of nodes with different power levels toward the energy centric grouping in the whole network architecture. This is computationally expensive and negatively impacts the energy saving capability with scaled underwater networking scenarios.

On the other hand, nodes placed on the bottom of NSN identify its location locally (i.e., depth information), then, the route cost based on residual energy and TM can be calculated. This technique results in overall network performance and greener communication considering higher energy resources and dedicated operation areas without creating communication overheads in the overall underwater network environments [31]. Each supernode is represented as a particular sensor node with unique features such as high energy. It forwards data from sensor nodes to the sink with different power-level transmission. The supernodes are employed at different depth-levels at the top of the progress area to collect the data from ordinary sensor nodes and relay them to the sink. Ordinary nodes forward the data packets to one of the supernodes based on a multi-level depth structure.

The significant contributions of this work are as follows.

- In addition to supernodes, we propose a new depth-based network architecture for UWSNs. Unlike typical UWSN architectures, the proposed architecture can be used to avert the communication void by utilizing one-layer among supernodes.
- Moreover, we also divided TM2RP based on a multi-level depth distribution structure. Data packets are simply transmitted from the sender toward the sink node according to the depth-level number assigned to each sensor node by supernodes. This approach can reduce the number of hops involved in the forwarding procedure, reduce the total energy consumed, and further improve the network lifetime.
- In TM2RP, data packets are forwarded from the source toward the destination using selected nodes based on depth, link quality, and residual energy.
- The proposed architecture is assessed comparatively with state-of-the-art techniques considering energy-centric green computing metrics for underwater networking.

The rest of this paper is organized as follows. Section 2 revises some well-known routing algorithms in UWSNs by classifying them into non-localization and localization-based. Section 3 presents TM2RP. Section 4 addresses the comparative performance evaluation of TM2RP. Finally, Section 5 concludes the approach proposed in this paper with future research directions.

## 2. Related Works

In this section, we represent different routing algorithms used in UWSNs related to the proposed work. A classification of the two main categorizations, algorithms using localization and algorithms without using localization, will be discussed with their drawbacks.

### 2.1. Algorithms Using Localization

In the context of algorithms using localization, it is expected that each sensor node has location information about itself and sinks using GPS that helps to identify the next forwarding nodes toward the sink. The critical point of all localization algorithms is to use location information for the forwarding process. However, it has significant variations in its method of identifying the best sensor toward the sink. Algorithms using localization look at different shapes between the sink and sensor node to determine the next forwarding nodes such as cone, layer, virtual pipeline, and zone toward the sink. The following paragraphs discuss some well-known algorithms using localization.

The authors in [32] proposed a localization routing method named the vector-based forwarding protocol (VBF). The presented approach focuses on solving the problems in the UWNSs (i.e., robustness, scalability, and energy-efficiency). The VBF protocol selects the next nodes based on using fix virtual pipeline from the sender toward the destination. Any sensor node located inside the virtual pipeline is elected as a next-hop, and the rest nodes are discarded. Nevertheless, VBF suffers from significant drawbacks. The full positioning information is required for each sensor node and the performance is influenced by the unavailability of sensors at the predefined radius in sparse networks.

The authors in [33] provided a new version of VBF by developing hop-by-hop vector-based forwarding (HH-VBF). This scheme utilizes a simulated pipeline from the sender node to the sink with a fixable pipeline regarding the location of the nodes that is improving the process of electing the next-hop node. Each sender node initializes a pipeline independently to send the data packet. Like VBF, the delivery ratio is increased, and energy consumption is dramatically decreased. Furthermore, the employment of positioning information becomes the core drawback of HH-VBF because of the inevitability of GPS in underwater areas.

In addition, vector based void avoidance (VBVA) is another improvement of VBF that employs the principles of HH-VBF regarding electing the next-hop nodes without using energy nor reliable parameters [34]. The main variance between VBF, HH-VBF, and VBVA is that VBVA employs dual 3-D flooding techniques to avoid the empty areas, named vector-shift and back-pressure.

Another routing protocol called focused beam routing (FBR) on a cross-layer location-based has been presented by [35]. The FBR protocol selects the next forwarding node by utilizing different transmission power-levels. In the routing process, the sender sends a Request to Send (RTS) data packet to the neighbor’s transmission power-level. Whenever the sender receives the Clear to Send (CTS) packet from the neighbor, the data packet will send to the source of the CTS packet. In case the sender does not receive any CTS from its neighbor, the transmission power-level is raised to the next power-level. This process is frequently repeated until the CTS message is received successfully. However, the frequently repeated process and exchanging the RTS and CTS messages will lead to delay. Furthermore, the FBR suffers from the localization problem.

On the other hand, a non-location routing protocol has been conducted by [36] named the power-efficient routing protocol (PER), taking the energy consumption as the main contribution. The basic idea is that the sink node is positioned in the midpoint area, the rest nodes are deployed randomly in the transmission area, and the sender is placed at the bottom level of water. Based on the calculated angle between neighbor nodes, residual energy, distance to the sink, and the source node selects the optimal two neighbors.

### 2.2. Algorithms without Using Localization

Algorithms without using localization do not employ full location information to select the next forwarding nodes. In this category, routing algorithms use different information and criteria than localization algorithms to identify the next forwarding nodes such as hop-count, dynamic address, physical distance, depth, and layering. Based on these criteria, algorithms without using localization are divided into two groups: bacon-based and pressure-based. Beacon-based algorithms, during the routing process, identify the next forwarding nodes using specific information such as physical distance and ID distribution. The sink node has provided this information by sending beaconing messages to the network. In the second group, pressure-based algorithms employ depth information to identify the next forwarding sensor nodes using a pressure sensor that measures the depth locally without using any beacon message. Depth information helps the sender node to identify the shallower sensors among neighbors. The following paragraphs discuss some well-known algorithms using localization.

This section reviews the non-localization algorithms, which do not need full location data related to UWSNs from the sink. Authors in depth-based routing (DBR) proposed the first non-localization routing scheme [28]. Each node with a lower depth than the sender node can join the forwarding procedure; furthermore, all the nodes hold the packets for a specific time to keep the data packets. Specifically, the sensor with a lower depth becomes the shorter holding time. DBR uses depth only for forwarding the data. Consequently, each node has a smaller depth consistently included in the forwarding procedure. Thus, many nodes will die earlier than others, therefore, unbalancing the power consumption between different nodes and making routing holes (communication void) in the UWNs. Moreover, the number of repetitive transmissions grows with the rise in the network density due to the tiny variations between the nodes’ depth.

In terms of beacon-based techniques, in [37], the authors proposed a hop-by-hop dynamic protocol named H2-DAB as a non-localization protocol. Each sensor has a unique ID called (Hop-ID); this ID is indicated to all sensors based on hop count toward the sink node. The H2-DAB broadcasts a tiny message from the sink. The node that collects the message will get a Hop-ID. Then, the nodes will rise and broadcast a Hello message with a new Hop-ID. Each node placed near the sink will get a small Hop-ID because the Hop-ID increases with higher depth sensors. Accordingly, the sensor with a lower Hop-ID is selected to be a forwarding node. However, the H2-DAB protocol suffers from many issues such as the nodes with small Hop-ID die soon because these nodes are used continuously more than the rest nodes. Moreover, the H2-DAB applies on the matric method that is hop count based, which is not a proper UWSN approach. Moreover, inquiry requests during the forwarding process and inquiry reply messages inundated more delay and power consumption.

In [38], the authors introduced another location-free protocol based on physical distance named a reliable and energy efficient routing protocol (RERP2R). The proposed protocol is an extended form of the energy-efficient routing protocol for UWSNs [39]. The proposed approach intends to enhance the reliability between nodes, which can be done through choosing the next forwarding, depending on the link quality. Therefore, the RERP2R employs a link quality metric called a physical distance and residual energy as well as expected transmission count (ETX). RERP2R consists of three stages: initialization, packet forwarding, and, finally, the maintenance stage. In the first stage, sensor nodes exchange the residual energy between neighbors and measure the ETX along with the distance. Next, each sensor spreads these data to its neighbors. Finally, all nodes obtain information about their neighbors’ residual energy, ETX, and physical distance. In the second stage, the node chooses the next-hop sensors close to the sink with excellent link quality and high residual energy. In the final stage, the physical distance is redetermined by sending tiny messages after a specified period, resulting in increased energy consumption. Furthermore, the ETX estimator’s use is not practical as it only uses one metric to measure the link quality, the packet reception rate [40,41].

In [42], a new non-location multi-layered routing protocol (MRP) was presented using supernodes. The supernodes are linked to the sink located on the water surface, whereas the regular nodes are scattered at the water’s bottom level. On the other hand, supernodes are designed with high energy and different power-level transmission. These nodes are employed for synchronization and obtaining the locations of the nodes in the network. MRP is categorized into two stages: layering and forwarding. In the first stage, supernodes broadcast beacon messages with different power-level transmissions, respectively containing the layer ID and its ID. The nodes that receive this message will assign this layer’s ID and the supernodes’ ID and record this information. After that, the power-level is increased and broadcasts the layer ID and supernode ID to the nodes. This procedure is repeated until all sensors are assigned with a layer and supernode ID. In the second stage, the source node sends the data packets using the layer-to-layer technique; each node receives the data packet computed, holding time based on residual energy. These nodes hold the data packet for a specific time to avoid duplicate packet transmission. If the holding time for one of the nodes is finished, it directly transmits the packet using the flooding technique to the next layer (next layer ID). All nodes that receive the same data packet discard these data. The data packet then reaches the supernode, and the supernode transmits it to the sink with maximum power transmission.

To conclude, both the MRP and H2-DAB protocol employs the same layering approach. Furthermore, Hop-ID in H2-DAB and layer ID in MRP is mostly the same. MRP obtains more reliable links than H2-DAB. Nevertheless, MRP undergoes some obstacles. The supernode utilizes maximum power to broadcast the message to another supernode placed at a high level, resulting in packet collision in multiple packets transmission and requires resending the data packets by the sensor node. Therefore, it occurs in high energy consumption. Furthermore, the MRP protocol ignores the link quality parameter, which affects the packet reception rate.

In this paper, the depth metric was employed to differentiate the depth level of neighbors. TM2RP is dissimilar and valuable from DBR, EEDBR, and MRP as follows.

- DBR utilizes the depth metric to choose the next hop sensor nodes, whereas EEDBR employs residual energy to choose the next-hop nodes. In contrast, TM2RP utilizes residual energy and link quality to choose the efficient link among neighbors.
- MRP utilizes only supernodes and residual energy to choose the next-hop nodes, whereas TM2RP utilizes supernodes to create a different level and choose the next hop using residual energy and link quality to reduce the elected sensors.

## 3. Triangle Metric Based Multi-Layered Routing Protocol

This section presents the routing protocol TM2RP in detail including network architecture and all phases executed in our routing protocol.

### 3.1. Network Architecture

**Figure 1:**
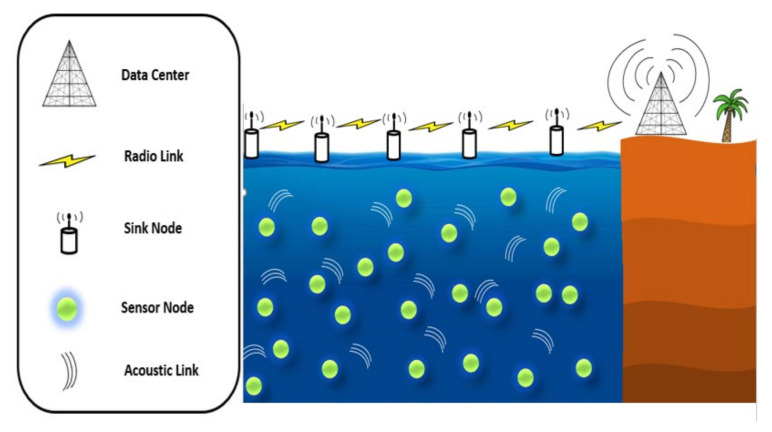
Traditional underwater sensor network architecture.

Figure 1 indicates the popular UWSNs’ structure utilized in well-known protocols. Sink nodes are organized on the water top linked by radiofrequency hyperlinks. All regular sensor nodes are deployed randomly underwater from top to bottom at different depths and assumes that those sink nodes are applied with acoustic and radio links. Sensor nodes forward the data packets from the source node toward the sink/destination via forwarding the data packets using sensor nodes positioned closer to the sink. Individually, the sensor nodes deployed at the lowest of the deployment area may forward the data packet in a hop by hop manner toward the sink. On the other hand, the sensor nodes must be available at various depth levels for data transmission. However, due to the node’s movement, there is a high chance of expanding a void region in the network, leading to a communication void problem (i.e., there is no available node with positive progress toward the destination to forward the data packets). Furthermore, we agree that reducing the number of information exchange between nodes significantly impacts decreasing network overheads and increasing communication throughput. It is highlighted that the proposal is a step closer to reducing information exchange in underwater networking by focusing on supernode consideration in the networking scenarios. These nodes have more information surrounding their local network environment, resulting in a lesser number of hello packet information exchanges in the overall network environment. This technique further reduces energy consumption toward green computing scenarios in underwater networking environments.

**Figure 2:**
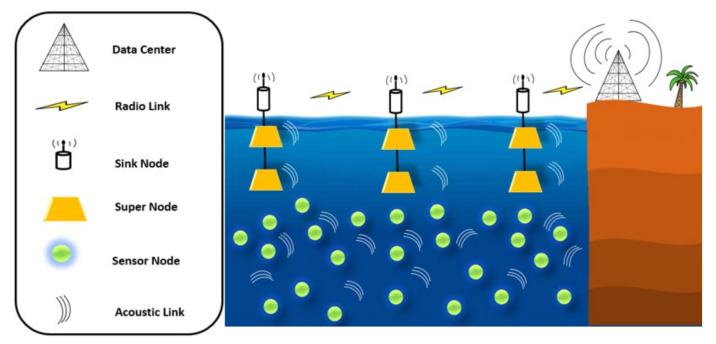
The proposed supernode layering based underwater network architecture.

The channel mode of TM2RP is focusing on accurate calculation of signal spreading and total noise loss for underwater networking environment. It has basically extended the channel presented in [43] considering the signal attenuation and absorption loss rate for underwater acoustic signal. The absorption loss $\alpha \left(f\right)$ of an underwater communication channel can be computed as expressed in Equation (1).

(1) $$10\log\left(\alpha \left(f\right)\right)=\left\{\begin{array}{c} \frac{0.11f^{2}}{\left(1+f^{2}\right)}+\frac{44f^{2}}{\left(4100+f\right)}+2.75\times 104f^{2}+0.003,\;f\geq 0.4\\ 0.002+0.11\left(\frac{f}{\left(1+f\right)}\right)+0.011f,\;f<0.4 \end{array}\right.$$

where f represents the frequency of the acoustic channel. The absorption loss is measured in dB/km and channel frequency in kHz. The absorption loss value α can be computed as $\;\alpha =\frac{10^{\alpha \left(f\right)}}{10}$. By combining the absorption loss and spreading loss, the total attenuation $A\left(l,f\right)$ can be expressed as follows in Equation (2).

(2) $$10\;\log\left(A\left(l,f\right)\right)=k\times 10\log l+l\times 10\log\left(\alpha \left(f\right)\right)$$

where l denotes the distance at which spreading loss is computed as represented by the first part of the right side of this equation, and the second part is denoted by the absorption loss at frequency f. Additionally, the underwater signal propagation geometry is denoted by the spreading coefficient k, which is in the first part of this equation. Therefore, we proposed an improved network architecture than above-mentioned for underwater networking. The supernode has been presented in MRP [42] to identify different layers with different power levels toward the whole architecture. Figure 2 below shows the improved architecture of the proposal with two different sensor nodes called ordinary nodes and supernodes. In this architecture, supernodes are embedded with high energy and high ability packet transmission with different power-level. Supernodes are static and positioned at the bottom level of the water surface. Supernodes have multi-use in our protocol, as discussed in next subsection. This architecture helps reduce the void areas in the network and avoids the communication hole. Moreover, sensor nodes have limited energy. Therefore, the nodes’ energy is soon exhausted. Therefore, utilizing supernodes can help in reducing energy consumption and further optimize the network lifetime.

It is highlighted that we utilized a supernode to identify one-layer among the supernodes. The nodes placed in the identified layer can forward the data packet directly to the supernode, more precisely, supernodes responsible for determining the NSN. On the other hand, nodes placed on the bottom of NSN identify its location locally (i.e., depth information). The residual energy and TM are utilized to calculate the route cost for underwater communication networking. This technique results in overall network performance and greener communication considering higher energy resources and dedicated operation areas without creating communication overheads in the overall underwater network environments. It is also highlighted that anchor and beacon node-based approaches lead to less energy consumption and overhead communication reduction in terms of beaconing messages. This is very well presented considering 3D localization in wireless networking [44,45]. However, the supernode based approach is about deploying special nodes for particular purpose operations, which results in overall network performance and greener communication considering higher energy resources and dedicated operation area in the network without creating communication overheads in the overall underwater network environments.

It is highlighted that the non-location-based consideration is a step closer toward reducing information exchange overhead in underwater networking by focusing on supernode consideration in the networking scenarios. Theses nodes have more information about the surrounding local network environment resulting in a lower number of hello packet information exchange in the overall network environment. This further reduces energy consumption toward green computing scenario in underwater networking environments. Regarding neighbor node consideration, we want to clarify that the accurate neighbor node information helps in making better forwarding decisions. For example, in the case that a higher number of neighbor node lesser packets should be transmitted considering duplication probability, whereas in the case of sparse scenario with lower number of neighbor nodes, more packets might need to be transmitted for increasing reachability probability.

### 3.2. Link Quality Centric the Triangle Metric

The underwater acoustic communication environment is highly constrained considering the bandwidth limitation, lower speed, and higher propagation delay. Therefore, any retransmission attempt during acoustic communication significantly increases the overall network communication systems’ energy consumption and reduces the network lifetime. Toward energy-oriented reliable acoustic communication link selection, link quality estimation becomes the first-class standard directly affecting the overall lifetime of the underwater network. For the traditional wireless networking environment, measuring expected transmission count (ETX) is the preferred choice for link quality estimation [46]. Furthermore, ETX-based link quality estimation has also been utilized in underwater literature to integrate local knowledge of underwater networking environment with count-based link quality [38]. However, many transmission requirements for link quality estimation make it less feasible for an acoustic network environment.

However, in this proposal, a layered underwater networking environment was considered where a multi-level pressure-based routing algorithm that estimates the link’s appropriateness considered the pressure-centric layering of overall acoustic networking scenarios. Considering dynamic underwater, we want to highlight that triangle metric (TM) oriented link quality estimation is considered an underwater networking centric improvement of expected transmission count (ETX)-based link quality estimation in traditional wireless networking. The TM-based link quality estimation’s vital benefits include reaping a brief assessment, reducing overheads, and a reliable estimation of acoustic links in an underwater scenario. The TM is a geometric normalization of three parameters including packet reception rate (PRR), signal-to-noise ratio (SNR), and link quality indicator (LQI). TM is a more stable and shorter overhead centric link estimation in underwater. Furthermore, it considers proper normalization between dynamic and static environments crucial for underwater networking [47].

As TM-based link estimation is a mixture of SNR, PRR, and LQI, so one can assure dependable and speedy link first-class estimation via calculating the mean SNR and LQI. The main formula of the TM can be defined as n packets, and m total packets received successfully, where (0<m<n). Moreover, LQI and SNR for each successful ${\mathrm{packet}}^{i}$ are denoted by $SNR_{i}\;and\;LQI_{i}$. The forwarder node broadcasts ten tiny packets in a short period to its neighbors. Upon receiving, the receiving nodes calculate the mean SNR and LQI using the two equations below:

(3) $$\bar{SNR}=\frac{\sum_{k=1}^{m}SNR_{k}}{n}$$

(4) $$\bar{LQI}=\frac{\sum_{k=1}^{m}LQI_{k}}{n}$$

The receiver node measures the distance between sensors (period of the hypotenuse) characterized through a point $\left(\bar{SNR},\bar{LQI}\right)$ and the point $\left(0,0\right)$ by the following equation based on Equations (3) and (4):

(5) $$\Delta d=\sqrt{{\bar{SNR}}^{2}+{\bar{LQI}}^{2}}$$

**Figure 3:**
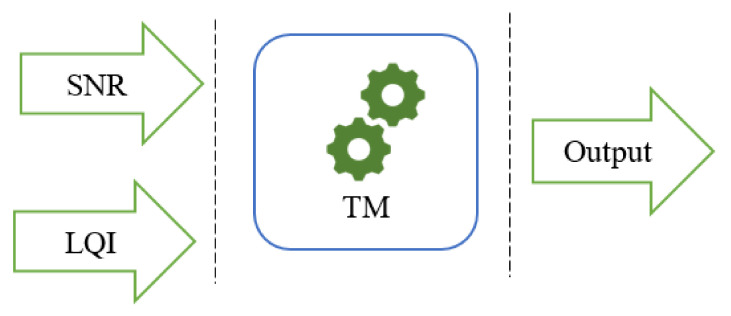
Triangle metric based link quality estimation in underwater networking.

The next step is the sender node, which measures the hyperlink for all of its neighbors as the highest Δd is the efficient link. A simple architecture of the TM-based link estimation is presented mainly through a three-phase-based estimation including SNR and the LQI centric input phase, TM processing phase, and the output categorization phase (see Figure 3). The link’s output categorization depends on threshold consideration for acceptable links in an underwater environment [48]. In our algorithm, every node estimates the quality of links based on the TM for its one-hop neighbors. The neighbor information table (NIT) is utilized for one-hop neighbor identification in the information acquisition phase. We want to highlight that LQI indicates the quality of the received signal computed using the energy detection (ED), the signal to noise ratio, or both. This measure is essential at the network layer as the routing metric can benefit from received signal strength (RSSI). RSSI gives the strength of the received signal. This is measured over the first eight symbols following the start delimiter of a frame. The estimation of RSSI and LQI is affected by some parameters such as power received packet, and background noise can be used to check the direction of RSSI and LQI. In summary, this proposal focuses on creating novel triangle metrics for green communication and computing in an underwater networking environment. We want to clarify that the TM algorithm is performed during the information acquisition phase, detailed below as the next sub-section.

### 3.3. Information Acquisition

**Figure 4:**
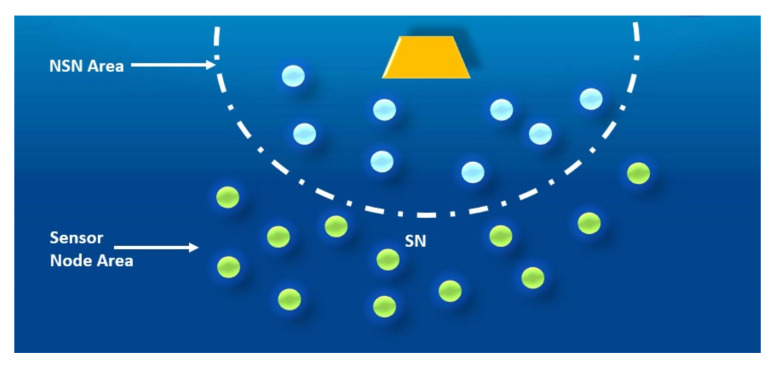
An illustration of assigning layer ID for neighbor of supernode (NSN).

In this stage, unlike MRP, we form one layer around the supernodes placed at the highest supernodes. Through this layer, the neighbor nodes of the supernodes are assigned with layer ID. The process of assigning this layer ID can be done as follows. The supernode placed at the highest depth broadcasts a tiny hello packet with transmission power level p1. This hello packet contains the layer ID (i.e., ID1). The nodes that receive this packet will be assigned as layer ID1, as shown in Figure 4. This critical point is that once we have data to send, these nodes will forward the data packets directly to the supernodes.

**Figure 5:**
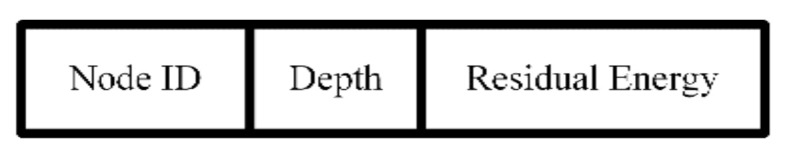
Hello packet format considered in the proposed underwater networking architecture.

Second, other sensors without IDs broadcast a hello packet to its neighbors including the node’s ID, residual energy, and depth, as shown in Figure 5. The sensor nodes then extract the depth embedded with the received packet and compare it with its depth. If the extracted depth is less than the node’s depth, the extracted information is stored in the NIT. Otherwise, it ignores the packet. Specifically, each sensor collects the primary information that helps in the forwarding process and store them in NIT. Next, each node then calls the TM algorithm to measure the Δd for nodes in the table, as discussed in the previous section by broadcasting tiny packets including node ID. Finally, the calculated distance is added to the NIT on the highest distance based on the highest rank.

At the end of this segment, NSN is assigned with ID. Each sensor node turns out to be aware of their elected neighbors’ information including residual energy, depth, and calculated distance.

Algorithm 1 describes the step by step process of executing the information acquisition phase in the routing framework. As shown in the algorithm, the first procedure GenerateProb (lines 1 to 8) is employed in a specific interval of time by SuperNode to generate a probe packet and broadcast it to its one-hop neighbors with power transmission level P1. Moreover, the second procedure ReceiveProb (lines 10 to 12) is called to check the probe packet and take the proper action based on the steps mentioned in previous paragraphs. The third procedure GenerateHello (lines 14 to 21) is also employed in a specific interval of time by all sensors ($node_{i}$) to generate a hello packet and broadcast it to its one-hop neighbors. Finally, the fourth procedure ReceiveHello (lines 23 to 34) is called to extract the hello packet and take the suitable action based on steps mentioned in previous paragraphs. In this procedure, TM has been called to calculate the link quality (line 30).

Due to the nodes’ movement in UWSNs, it is stated that the nodes’ location frequently changes. However, some nodes may join another node’s range, or some current neighbors may go out of node range. Therefore, the nodes that have been inserted in NIT should be updated. More specifically, the information acquisition phase is periodically called to update the NIT of each sensor node at a given time interval. At the end of this process, the NSN is identified, and each sensor node becomes aware of the information about their neighbors such as depth, residual energy, and TM-based on distance.
**Algorithm 1.** Information Acquisition Phase.1:
procedure
GenerateProb
(
SuperNode)
2: if ProbTimeout is finished then3:
  Generate
ProbPacket
4:   
Add id to ProbPacket
5:   
Broadcast
(
ProbPacket
)
6:   
Set
NewTimeout
7:   end if8:end procedure9:10:
procedure
ReceiveProb
(
$node_{i},\;ProbPacket$
)
11: 
$Add\;ProbPacket\;information\;to\;node_{i}.NIT$
12:end procedure13:14:
procedure
GenerateHello
(
$node_{i}$
)
15: if HelloTimeout is finished then16:
 Generate
HelloPacket
17: 
Add id, depth and residual energy to hello packet
18: 
Broadcast 
(
hello packet
)
19: 
Set
NewTimeout
20: end if21:end procedure22:23:
procedure
ReceiveHello
(
$node_{i},hello\;packet$
)
24:
 if
$\left|node_{i}.depth\right|>\left|hello\;packet.depth\right|$
then
25:
  if
hello packet ID
is not in
$node_{i}.NIT$
then
26:  
$Add\;hello\;packet\;information\;to\;node_{i}.NIT\;$
27:  else28:   
$Update\;information\;in\;node_{i}.NIT\;$
29:  end if30:  Call TM ($node_{i}$)31:  else32:
  Drop (
hello packet
)
33: end if34:end procedure

### 3.4. Data Forwarding

In this step, the sender’s data packets are transmitted toward the distention/sink, as shown in Algorithm 2. The next-hop node should be shallower and much closer to the destination, taking into account the best link quality and high residual energy. Sensors that are closer to the distention have a high chance of transmitting data packets. We estimated the route cost based on TM and residual energy to choose the next-hop node. Therefore, the calculation of route cost is estimated between sensors $\left(x,y\right)$ using Equation (6) below:

(6) $$RouteCost=\left(1-\frac{Re_{y}}{Re_{max}}\right)+\left(1-\;\frac{\Delta d_{\left(x,y\right)}}{\Delta d_{max}}\right)$$

where $Re_{y}$ is the residual energy of node y; $Re_{max}$ is the maximum energy of the node; $\Delta d_{max}$ is a pre-set metric (i.e., we select $\Delta d_{max}$ after different simulation scenarios); and $\Delta d_{\left(x,y\right)}$ link quality calculation between x and y. Equation (6) computes the route based on extraordinary parameters: link quality and residual energy. Primarily based on Equation (6), it is expected that the node located at a lower depth than the sender with satisfactory link quality and higher residual energy may have minimum path cost. Thus, this node could be decided on as an optimal node to transmit packets.

**Figure 6:**
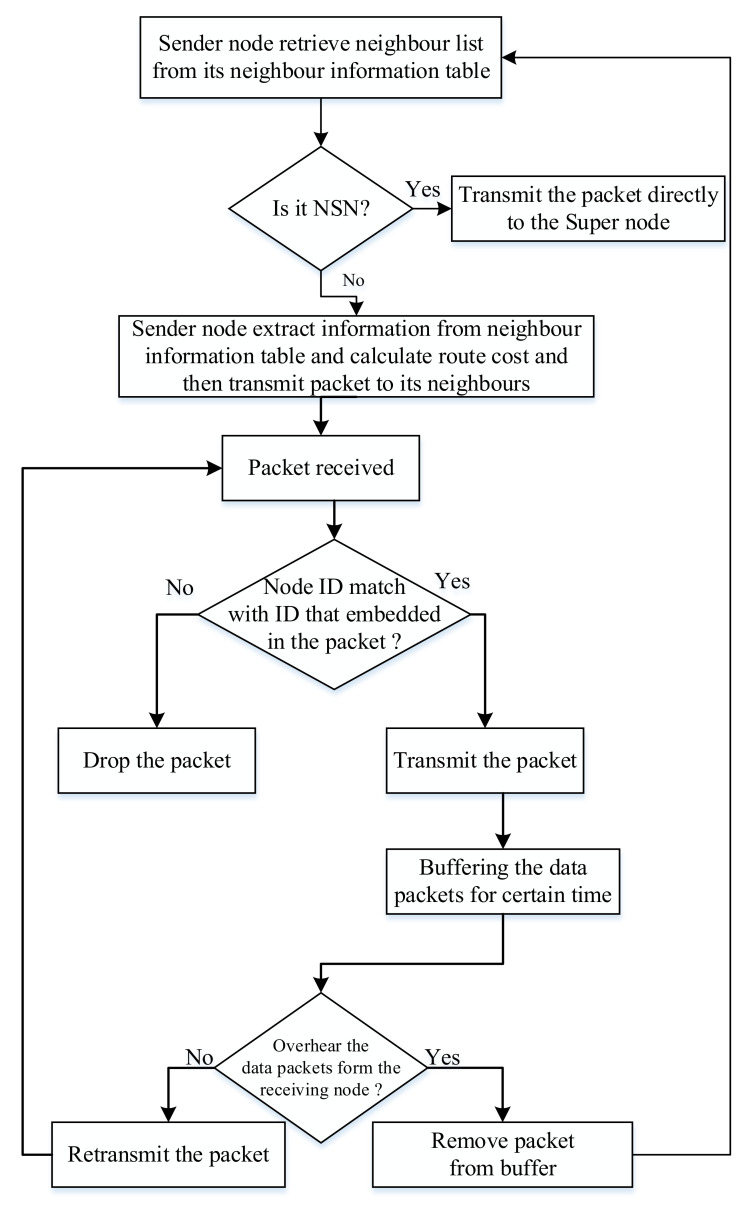
Flowchart of the underwater data forwarding phase in the proposed architecture.

In Figure 6, the forwarding procedure of the data packets can be concluded as below.

1. The sender checks if it is the NSN nodes by checking if it is assigned with layer ID, and it forwards the packet to the supernode.
2. Otherwise, it gathers information from the NIT and calculates the route cost based on Equation (6), as discussed above.
3. The sender chooses the next forwarding node with minimum routing cost.
4. The sender then embeds the selected node’s ID with the data packets.
5. The receiver nodes then compare ID within the data packet’s embedded ID. In this case, the data packet is only accepted by the nodes with the matched ID and ignored by other nodes. This process is repeated until the packet is received by the supernode.
6. The data packets are then forwarded to the sink.

**Algorithm 2.** Data Forwarding Phase.1:
procedure
MRPForwarding
(
$node_{i},\;data\;packet)$
2: if $node_{i}\;ID\;match\;forwarding\;ID\;in\;data\;packet$ then3:  Calculate route cost4:  Insert route cost into NIT5: else6:  Drop (data packet)7: end if8: 
Select the best node based on minimum route cost
9: 
Add ID to data packet
10: 
$node_{i}$
**buffers**
data packet
11: Broadcast(data packet)12: 
$node_{i}$
**Generates**
retransmission time
13: if $node_{i}\;overhear\;forwarded\;data\;packet\;$ then14:  Drop(data packet)15: else16:  Rebroadcast(data)17: end if18:end procedure

## 4. Results and Discussion

In this section, we discuss the experimental results of the proposed algorithm, then compare TM2RP with related routing protocols called DBR and MRP.

### 4.1. The Simulation Environment

In this part, we discuss the performance evaluation setting of TM2RP using the testing environment in the Aqua-Sim package for Network Simulator 2 (NS2) to implement the underwater networking scenario [49]. A random topology with a different number of sensor nodes was performed (i.e., 25–400) in the deployed area of $1250\;m^{3}$. The transmission range was assigned to 250 m, with an initial energy of 100 J and data packet generation time equal to 15 s for source node with data packet size equal to 64 bytes. A hello packet interval was set to 100 s. In this case, every 100 s, neighbor nodes were initiated with residual energy information for making forwarding decision. Next, distance based on the TM was calculated for prioritizing forwarding candidate neighbors. The energy model was utilized using the same as that used in VBF [32]. The power setting (2 w, 0.75 w, and 8 mw) represents the unit consumption level in data transmission, reception, and being idle at listening to the channel. We then employed a media access control (MAC) 802.11−DYNAV protocol and the result was averaged from 25 runs [50]. We want to highlight that the DYNAV protocol is a potential improvement of 802.11 for underwater networking scenarios. It focuses on dynamically calculating the network allocation vector (NAV) for individual nodes, resulting in significant performance benefits to avoid unnecessary loner communication deferring by neighboring nodes. Table 1 below lists the simulation sitting. We want to clarify that the initial energy level consideration in any simulation experiment majorly depends on the network size, transmission range, and data size consideration for communication. We believe that our consideration of 100 J of initial energy in each sensor node was suitable for our experiment’s network setting. We do agree that the realistic underwater sensor network is generally sparse in current application scenarios where the majority of the services are related to the normal monitoring of sea borders. However, when we think of next generation underwater smart and critical services such as underwater object recognition, micro mobility tracking, etc., the underwater sensor network should consider dense networking scenarios. Our consideration of a 250 m distance between sensor nodes is basically appropriate for such underwater networking applications.

### 4.2. Performance Metrics

We used the four main performance criteria tested in the most state-of-the-art routing algorithms in UWSNs as discussed below:

a. Energy consumption: the total amount of consumed energy by each sensor for the successfully forwarded packets.
b. Packet delivery ratio: the total ratio of the packet that reaches the distention/sink successfully to the number of transmitted packets by the source node.
c. Network lifetime: the overall lifetime of the networks calculated based on the first die node in the network.
d. End-to-End delay: the average delay between sensors used to forward the packets toward the destination.

For experimental implementation clarification, our protocol is developed using a C++ programming language in the network layer in the Aqua-Sim package as a formal routing protocol for the ns2 network simulator. Furthermore, different underwater simulation scenarios have been developed using the TCL programming language. After executing the protocol with different underwater network scenarios, the results were extracted from trace files using a scripting language to save the results in a standard text file. The figures presented in the result section are a graphical representation of the text file data.

### 4.3. Analysis of Results

**Figure 7:**
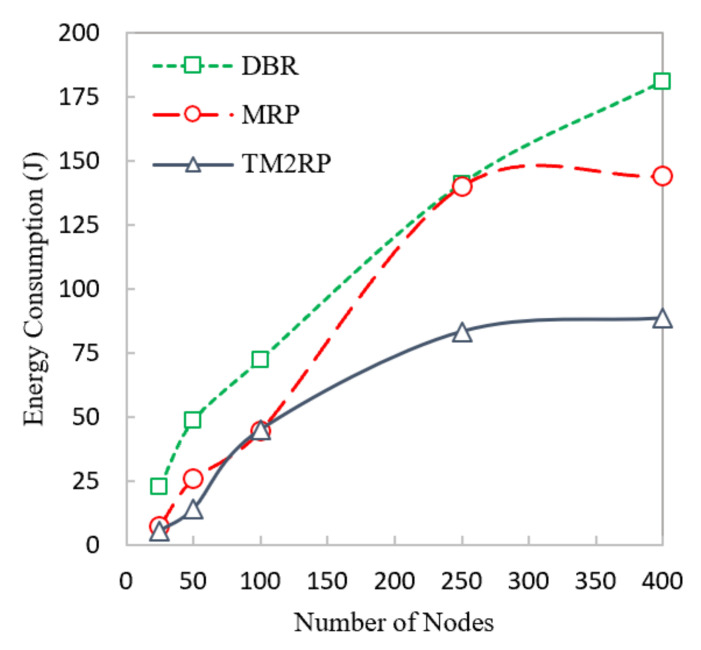
Comparison of underwater network energy consumption with number of sensor nodes.

This section provides a comparative analysis of the proposed protocol with existing schemes. Energy consumption for DBR, MRP, and TM2RP were measured in this section, and the results are shown in Figure 7. DBR did not employ an energy metric. It utilizes depth metric, which directly impacts the energy consumption that is consciously reduced, leading to consuming high energy. Moreover, MRP employs the residual energy and depth metric for selecting the next forwarding, which consumes less energy than DBR. Furthermore, the use of supernodes in MRP reduces the number of packet transmissions. In contrast, TM2RP has more energy balancing compared to DBR and MRP. This is because the use of residual energy with a link quality metric in TM2RP reduces the energy consumption between nodes considering the quality of the link, leading to choosing the forwarder node toward the distention efficiently. Therefore, TM2RP accomplishes low energy consumption compared to MRP and DBR.

**Figure 8:**
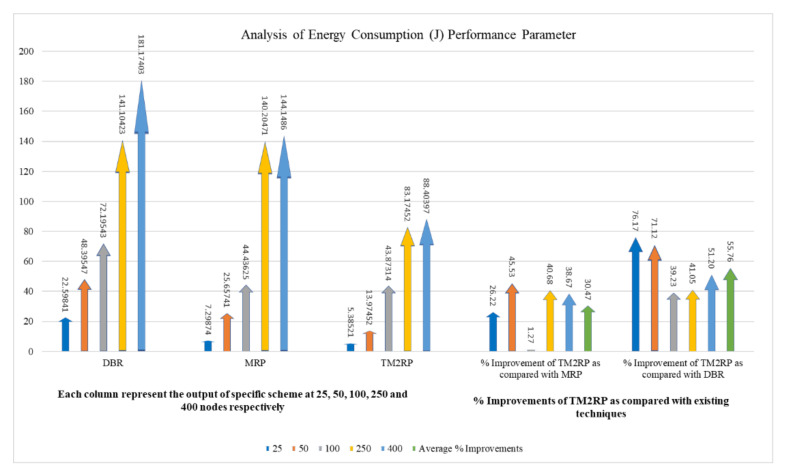
The statistical outcomes of the comparative protocols with regard to energy consumption.

The proposed protocol’s total energy consumption is investigated in detail in Table 2, compared with existing techniques focusing on the respective average obtained values. It shows that the average energy consumption values obtained by TM2RP were 30% and 55% for MRP and DBR, respectively. Based on the percentage above, the proposed algorithm provides more stability compared with the literature. TM2PR employed underwater characteristics resulting in high energy-saving performance. This result is further shown clearly in Figure 8, where energy consumption observations and percentage gain are demonstrated in close relation for better clarity. The results provided in Table 3 and Figure 8 are thus confirmed. Therefore, with significantly lower energy consumption, the proposed protocol outperformed state-of-the-art algorithms.

**Figure 9:**
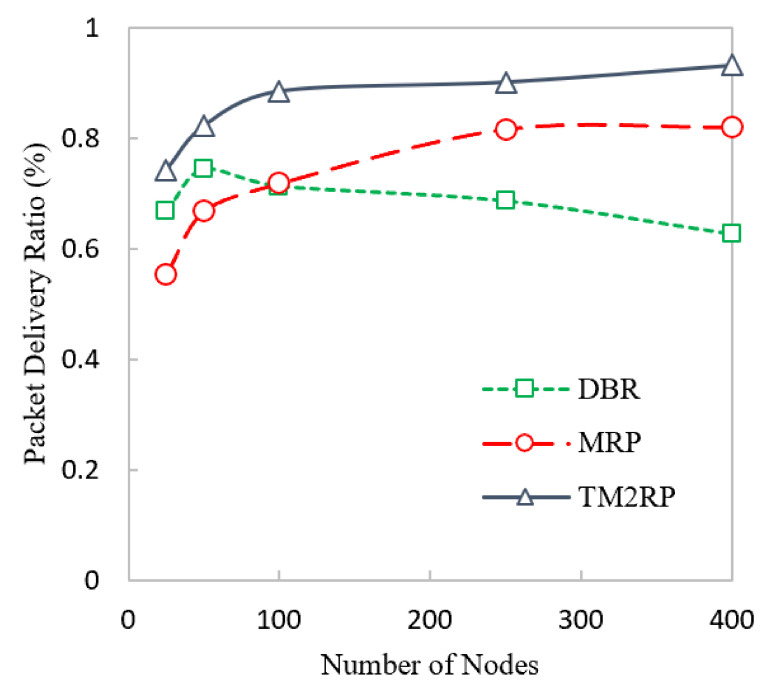
Comparison of underwater packet delivery ratio with increasing number of nodes.

The packet reception rate for DBR, MRP, and TM2RP is indicated in Figure 9. Selecting the next forwarder in DBR using depth information makes the data packets transmit using the same nodes, reducing packet loss, and resulting in the high delivery ratio in DBR compared to MRP with a smaller number of nodes. Moreover, MRP obtained less delivery ratio with less energy consumption than DBR because it provides energy balancing, but does not employ link quality metrics. In contrast, TM2RP achieved the highest packet delivery ratio using less expensive methods. Therefore, the employment of retransmission techniques reduces the packet loss if the sensor has not been selected to transmit the data packets, which leads to an increased packet reception rate.

We agree that link quality calculation is significant for the proposed framework. As much as link quality calculation or prediction will be better, the performance of the proposed framework is improved with regard to the packet delivery ratio. This is due to the average value consideration of LQI in Equation (4). Thus, the average value closeness to the actual value will impact the overall selection of underwater forwarding nodes resulting in a high packet delivery ratio compared to state-of-the-art techniques. Furthermore, TM2RP utilizes the TM, which obtains more reliable and stable links, which reduces packet loss and results in a high delivery ratio toward the supernodes.

**Figure 10:**
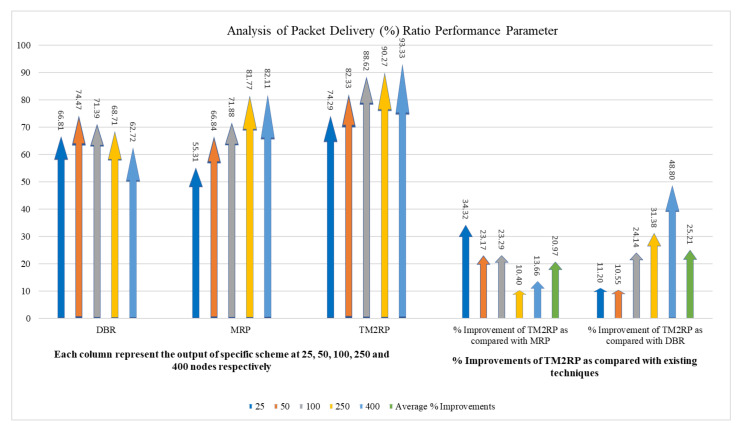
The statistical outcomes of the comparative protocols with regard to packet delivery ratio.

A more detailed performance gain analysis is provided in Table 3 in terms of the packet delivery ratio of TM2RP. This comparative study investigated state-of-the-art underwater literature to illustrate the corresponding analysis of performance gain. It can be noted that in terms of percentage, the average values obtained by TM2RP were 20% and 25% for MRP and DBR, respectively. The justification behind this is the use of underwater features to distinguish network movements that have not been studied in any current research. The existing algorithms use the quality of service and the location of the underwater cluster heads to select the next forwarding nodes. However, the proposed system addresses the underwater network complexities, accomplished by better results regarding the packet delivery ratio. This result is illustrated in Figure 10 in a more technically comprehensible manner. Here, in close contrast, the average values obtained results by TM2RP are provided to make it readable compared to the literature. This is also valuable for validating the outcomes shown in Table 2, and Figure 9 demonstrates that TM2RP outperformed other techniques in terms of packet delivery ratio.

The overlapping in Figure 7 and Figure 9 was due to lower performance difference between the literature protocols under smaller network scenario with limited number of sensor nodes. In particular, the energy consumption performance of MRP and the proposed TM2RP was quite close with a smaller underwater network of less than 100 underwater sensor nodes. This is due to the individual node’s residual energy and depth information-based strategy of MRP, which works with small network environment. However, the energy consumption performance considerably degrades with a scaled network environment of 150 or more underwater sensor nodes. Similarly, the packet delivery ratio performance of DBR and MRP was quite close with smaller network scenario and the same with 100 sensor nodes because of the similar depth-based forwarding approach without creating layers, which works precisely with a smaller number of nodes.

**Figure 11:**
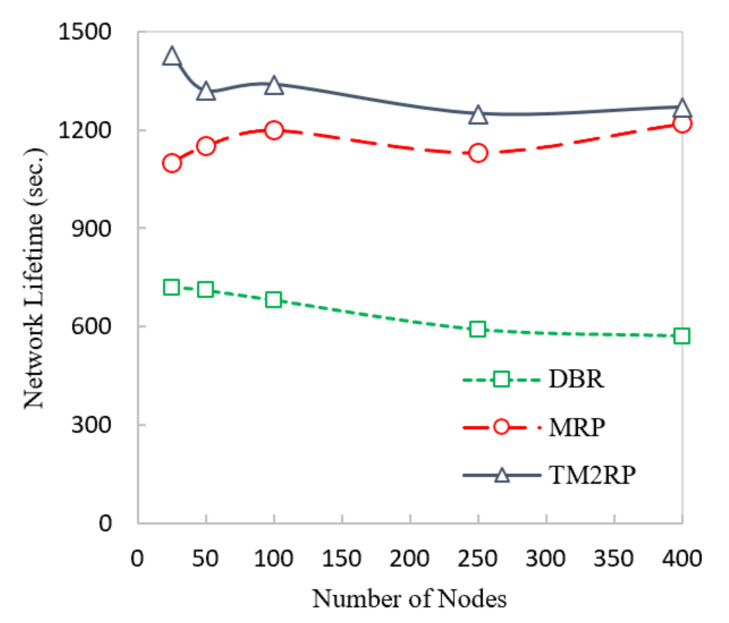
Comparison of underwater network lifetime with increasing number of nodes.

Figure 11 below shows the comparison of network lifetime between DBR, MRP, and TM2RP. In this figure, DBR and MPR have a lower network lifetime than TM2RP. DBR utilizes depth information only, leading to selecting a lower depth, always ignoring the node’s energy. As a result, the selected node will die soon, and the network lifetime is further reduced. In MRP, depth information with residual energy has been employed for selecting the next forwarding nodes, meaning then that the node with high residual energy has always been selected. Moreover, other nodes have not been selected, which directly impacts on the network’s lifetime. On the other hand, TM2RP calculates the route cost using link quality, residual energy, and depth information. In this case, the energy consumption has been balanced and further improves the network lifetime.

**Figure 12:**
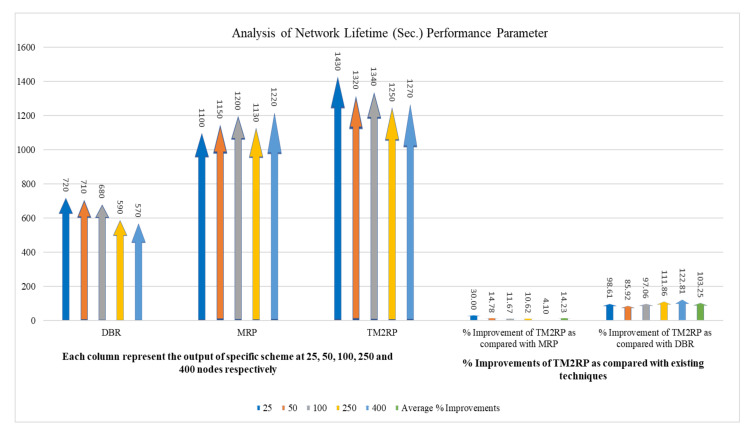
The statistical outcomes of the comparative protocols with regard to network lifetime.

In Table 4, the network lifetime TM2RP is investigated compared to the literature in terms of percentage gain. The average values obtained by TM2RP can be noted as 14% and 103% for MRP and DBR, respectively. The performance strengths can be explained by the lack of utilizing the underwater features in the existing algorithms, depending primarily on the quality of service and the position information of the sensor nodes. However, underwater features have been considered in the proposed protocol, resulting in significant efficiency gains as the network lifetime is longer. These obtained results in network lifetime are further represented in detail in Figure 12. Thus, network lifetime outcomes and observations are displayed in the deep association as it is comparatively analyzed. Consequently, it validates the outcomes displayed in Table 4 and Figure 11. Hence, in comparison with other state-of-the-art techniques, the proposed TM2RP indicated a more extended network lifetime.

**Figure 13:**
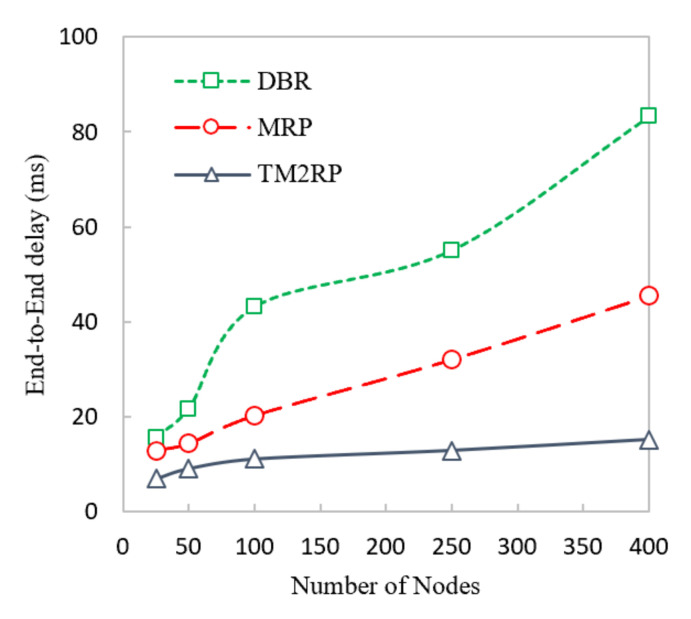
Comparison of underwater end-to-end delay performance with increasing number of nodes.

The average end-to-end delay of TM2RP, DBR, and MRP is measured in the following figure. Figure 13 proves that TM2RP achieved a lower delay than DBR and MRP. This is because DBR and MRP employ holding time based on residual energy. This holding time resulted in increasing the delay if all neighbors did not overhear and was acknowledged from its neighbor. In this case, the second elected node forwards the data after the holding time is finished. As a result, the delay occurred. On the other hand, TM2RP did not employ the holding time technique as the electing node directly forwarded the data packet. This is because the sender node chose the receiver node. Only the sender node holds the data packet for a second until acknowledged by its neighbor, resulting in less delay than DBR and MRP.

**Figure 14:**
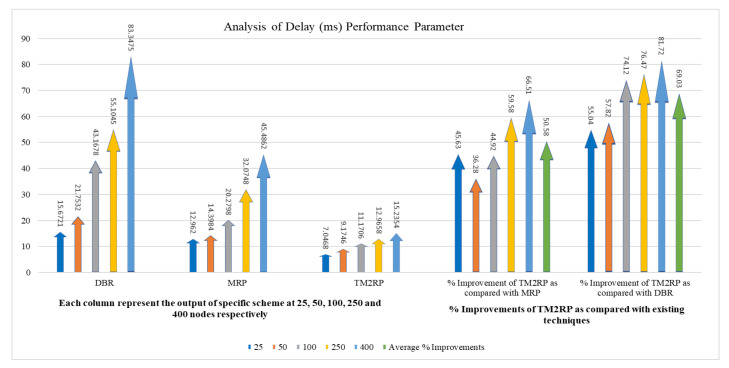
The statistical outcomes of the comparative protocols with regard to end-to-end delay.

A more comprehensive discussion of the end-to-end delay average obtained results of TM2RP is given in Table 5. For MRP and DBR, respectively, the average obtained results by TM2RP were 50% and 69%. This can indicate that the underwater characteristics are not considered in existing algorithms, depending on the location information of sensor nodes. On the other hand, TM2RP employed direct forwarding of the data packet without holding time, leading to significant results. This feature is demonstrated in Figure 14 clearly, where observations of the average obtained results of end-to-end delay are displayed comprehensively. The outcomes provided in Table 5 and Figure 13 were verified and demonstrate that TM2RP overwhelmed other state-of-the-art techniques regarding the end-to-end delay.

## 5. Conclusions and Future Work

In this paper, we proposed a triangle metric based multi-layered routing protocol for UWSNs, called (TM2RP). The proposed algorithm is at a pressure routing algorithm that employs supernodes, link quality, and residual energy to choose the efficient next forwarding sensor among its candidate. Residual energy has a direct impact on balancing the total energy consumed by sensors. The link quality contributes to identifying the most reliable links between sender and receiver nodes that optimizes the delivery ratio toward the supernodes. Thus, TM2RP combines these factors to calculate route cost. This route cost is then used to choose the next forwarding nodes. In the end, TM2RP employs an overhead and retransmission mechanism to suppress redundant packet transmission and avoid packet loss.

We evaluated the performance of TM2RP using the NS2 Simulator with Aqua-Sim package for underwater along with a comparison against DBR and MRP. Based on the simulation results, TM2RP achieved better results than DBR and MRP in terms of network lifetime, packet delivery ratio, energy consumption, and end-to-end delay. We suggest modifying the triangle metrics in future perspectives to combine more link quality metrics with improving stability and reliability links [23]. Moreover, the communication void in UWSNs and working in designing a void shows an awareness that TM2RP needs further investigations [51]. Next, selecting the shortest path is another crucial issue that is taken into account for designing the shortest path TM2RP [26,52].

## Figures and Tables

**Table 1:** The underwater network simulation parameter sitting.

Simulation Parameter	Value
Network topology	Random topology
Number of sensor nodes	25–400
Deployment area	$1250\;m^{3}$
MAC protocol	8.2.11−DYNAV
Bandwidth	10 Kbps
Area of transmission range	250 m
Hello packet interval	100 s
Communication medium	Acoustic Waves
Initial energy	100 J
Power consumption	2 w, 0.75 w, and 8 mw
Node movement	0–3 m/s
Packet generation time	15 s
Data packet size	64 bytes
Number of Runs	25

**Table 2:** The obtained results of TM2RP with regard to energy consumption.

Node vs. Energy Consumption (J)				% Improvements of TM2RP as Compared with Existing Techniques	
Nodes	DBR	MRP	TM2RP	% Improvement of TM2RP as Compared with MRP	% Improvement of TM2RP as Compared with DBR
25	22.59841	7.29874	5.38521	26.22	76.17
50	48.39547	25.6574	13.97452	45.53	71.12
100	72.19543	44.4363	43.87314	1.27	39.23
250	141.1042	140.205	83.17452	40.68	41.05
400	181.174	144.149	88.40397	38.67	51.20
Average % Improvements				30.47	55.76

**Table 3:** The obtained results of TM2RP with regard to packet delivery ratio.

Node vs. Packet Delivery Ratio (%)				% Improvements of TM2RP as Compared with Existing Techniques	
Nodes	DBR	MRP	TM2RP	% Improvement of TM2RP as Compared with MRP	% Improvement of TM2RP as Compared with DBR
25	66.81	55.31	74.29	34.32	11.20
50	74.47	66.84	82.33	23.17	10.55
100	71.39	71.88	88.62	23.29	24.14
250	68.71	81.77	90.27	10.40	31.38
400	62.72	82.11	93.33	13.66	48.80
Average % Improvements				20.97	25.21

**Table 4:** The obtained results of TM2RP with regard to network lifetime.

Node vs. Network Lifetime (Sec)				% Improvements of TM2RP as Compared with Existing Techniques	
Nodes	DBR	MRP	TM2RP	% Improvement of TM2RP as Compared with MRP	% Improvement of TM2RP as Compared with DBR
25	720	1100	1430	30.00	98.61
50	710	1150	1320	14.78	85.92
100	680	1200	1340	11.67	97.06
250	590	1130	1250	10.62	111.86
400	570	1220	1270	4.10	122.81
Average % Improvements				14.23	103.25

**Table 5:** The obtained results of TM2RP with regard to end-to-end delay.

Node vs. End-to-End Delay (ms)				% Improvements of TM2RP as Compared with Existing Techniques	
Nodes	DBR	MRP	TM2RP	% Improvement of TM2RP as Compared with MRP	% Improvement of TM2RP as Compared with DBR
25	15.6721	12.962	7.0468	45.63	55.04
50	21.7532	14.3984	9.1746	36.28	57.82
100	43.1678	20.2798	11.1706	44.92	74.12
250	55.1045	32.0748	12.9658	59.58	76.47
400	83.3475	45.4862	15.2354	66.51	81.72
Average % Improvements				50.58	69.03
